# Field-Free Spin–Orbit Torque Switching in Janus Chromium Dichalcogenides

**DOI:** 10.1021/acs.nanolett.4c03029

**Published:** 2024-09-13

**Authors:** Libor Vojáček, Joaquín Medina Dueñas, Jing Li, Fatima Ibrahim, Aurélien Manchon, Stephan Roche, Mairbek Chshiev, José H. García

**Affiliations:** † Université Grenoble Alpes, CEA, CNRS, IRIG-Spintec, 38000 Grenoble, France; ‡ ICN2 — Institut Català de Nanociència i Nanotecnologia, CSIC and BIST, Bellaterra, 08193 Barcelona, Spain; ¶ Universitat Autònoma de Barcelona (UAB), Bellaterra, 08193 Barcelona, Spain; § CEA, Leti, Université Grenoble Alpes, F-38054, Grenoble, France; ∥ CNRS, CINAM, Aix-Marseille Université, Marseille 13288, France; ⊥ ICREA — Institució Catalana de Recerca i Estudis Avançats, 08010 Barcelona, Spain; # Institut Universitaire de France, 75231 Paris, France

**Keywords:** spin−orbit torque, transition metal dichalcogenide, 2D materials, van der Waals ferromagnet

## Abstract

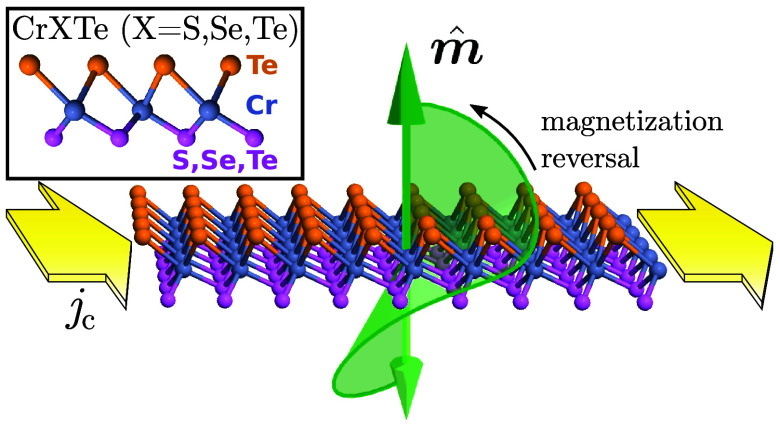

We predict a very large spin–orbit torque (SOT) capability of magnetic chromium-based transition-metal dichalcogenide (TMD) monolayers in their Janus forms CrXTe, with X = S, Se. The structural inversion symmetry breaking, inherent to Janus structures is responsible for a large SOT response generated by giant Rashba splitting, equivalent to that obtained by applying a transverse electric field of ∼100 V nm^–1^ in non-Janus CrTe_2_, completely out of experimental reach. By performing transport simulations on carefully derived Wannier tight-binding models, Janus systems are found to exhibit an SOT performance comparable to the most efficient two-dimensional materials, while additionally allowing for field-free perpendicular magnetization switching, due to their reduced in-plane symmetry. Altogether, our findings evidence that magnetic Janus TMDs stand as suitable candidates for ultimate SOT-MRAM devices in an ultracompact self-induced SOT scheme.

The spin–orbit torque (SOT) mechanism represents an innovative method to electrically manipulate the magnetization of a magnetic material,^1,2^ providing remarkable energy-efficiency, writing speed, and scalability prospects, which have earned their insertion in magnetic random access memory (MRAM) applications,^3,4^ among other developing technologies.^5−7^ SOT-MRAM prototype cells have been shown to operate on the subnanosecond time scale,^8−11^ with a power consumption of merely 1% of their (already in commercial use) spin-transfer torque counterpart.^12,13^ Although next-generation SOT-based technologies advance at a steady pace, they still face issues regarding massive density and integration. The development of SOT-MRAMs remains limited to multilayered devices, where SOT figures of merit are strongly sensitive to interface quality; while additionally, a densely packed SOT-MRAM requires electrical switching of magnets with perpendicular magnetic anisotropy (PMA),^14^ which is only achieved in conventional devices based on heavy metal/ferromagnet bilayers with the assistance of an external magnetic field.

van der Waals layered materials offer alternative paths to overcome these issues. Atomically clean interfaces have been shown to enhance both charge-to-spin conversion^15−18^ and tunneling magnetoresistance^19,20^ while reducing the cell dimensions. Furthermore, precise control of crystal symmetries enables novel SOT mechanisms that allow for field-free PMA switching.^21−24^ Materials such as CrTe_2_^25−28^ and Fe_*n*_GeTe_2_ (*n* = 3, 4, 5)^29−33^ offer the most promising alternatives to overcome the usually low Curie temperature (*T*_C_) of van der Waals ferromagnets (FMs), where additional gating, strain, and chemical composition engineering allow one to tune their magnetic properties.^30,34−36^ New avenues for SOT devices are opened when exploiting the metallic nature of these materials, along with their strong spin–orbit coupling (SOC), overcoming multilayer designs in favor of an all-in-one platform where the FM acts as both the SOC material and the free magnetization in a self-induced SOT scheme.^37−41^ In this context, Janus transition-metal dichalcogenide (TMD) monolayers stand out as the materials of choice.^42−44^ Indeed, the CrXTe ultrathin layers, similar to their non-Janus counterpart CrTe_2_,^45^ are expected to be magnetic with PMA under the adequate experimental conditions, high *T*_C_ even exceding room temperature, and are most stable in their metallic 1T phase.^46,47^ Inversion symmetry forbids an SOT response in CrTe_2_; however, this stepback is overcome by breaking the symmetry between both chalcogen atoms, for instance, by applying an electric field transversal to the crystal plane. Another symmetry-breaking mechanism is obtained by substituting one Te atom with S or Se, thus forming the Janus CrXTe structures, where broken inversion symmetry is manifest in the crystal field.

In this Letter, we predict an exceptional SOT performance of chromium-based Janus TMD monolayers CrXTe (X = S, Se), which allow for field-free switching of the perpendicular magnetic state, representing a qualitative improvement over other previously studied Janus TMDs.^43^ We deploy a robust end-to-end methodology, where starting from first-principles simulations we build Wannier tight-binding models which fully capture reciprocal space spin textures and perform quantum transport simulations and critical field-free PMA switching current calculations. We compare both the Janus and non-Janus materials under electric field, demonstrating that Cr-based Janus monolayers constitute an optimal SOT platform for low-energy magnetization reversal.

We perform first-principles calculations of the selected TMDs using density functional theory (DFT)^48,49^ with GGA-PBE pseudopotentials^50^ and an effective Hubbard *U* correction of 3.0 eV to localize the Cr-d orbitals.^51^ The usual metallic 1T phase, displayed at the center of Figure 1, proves to be more stable than the semiconducting 1H phase. Based on the maximally localized Wannier functions,^52,53^ we then derive tight-binding models of the DFT ground state. Accurate representations require a 22-orbital basis comprising the Cr-d and the chalcogen-p bands, with the interactions expanded into a 25 × 25 supercell. The ab initio band structure is thereby represented with an impressive ∼1 meV accuracy in a large window of approximately – 5 eV to +4 eV about the Fermi level. We also carefully derive the real-space spin operator in the Wannier basis,^54^ resulting in a nonlocal spin that replicates the ab initio spin texture with ∼3% error, ensuring a precise representation of the system’s symmetries (see sections S1 and S4 in the Supporting Information). Overall, the comparison between DFT results and those of the Wannier model yields an excellent agreement, as shown in Figure 1. This demonstrates the capability of our methodology, allowing to reach a DFT-level accuracy via tight-binding models for general systems.

**Figure 1:**
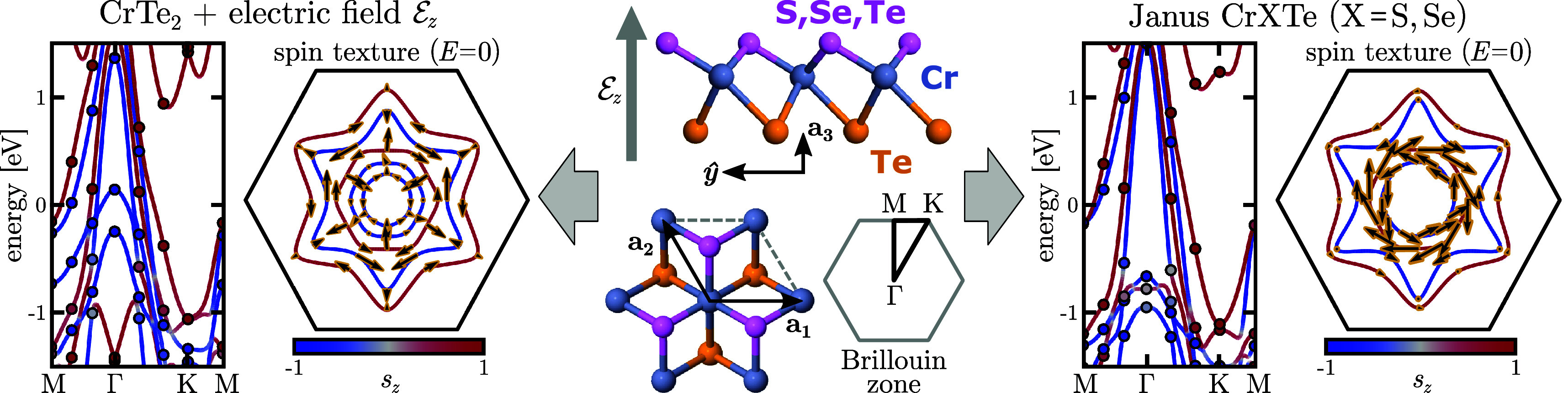
(Middle panel) CrXTe Janus structure, lattice vectors, and Brillouin zone. (Left panel) Band structure and spin-textures of symmetric CrTe_2_ under the effect of an applied electric field [ = 2 V nm^–1^]. The continuous band structure curves and black spin texture arrows are obtained from the Wannier model, while the discrete band structure dots and yellow highlighted spin texture arrows are obtained from DFT calculations. (Right panel) Band structure and spin textures of CrXTe [X = Se].

Note that the remarkable SOT capability of the low-symmetry Janus systems is already apparent from its Fermi-level spin textures, showing a prominent helical winding in CrXTe, displayed in the right panel of Figure 1. This large Rashba term comes from a strong inversion-symmetry breaking due to a huge internal out-of-plane electric field (∼1–2 V nm^–1^) caused by a charge imbalance at the two inequivalent chalcogen atoms S/Se and Te (see section S2 in the Supporting Infromation). In contrast, an external electric field  applied on CrTe_2_ to break its innate inversion symmetry is heavily screened due to its large dielectric constant ε_CrTe_2__, reducing any applied field by a factor of ε_CrTe_2__^–1^ ≈ 0.02 inside the layer. This is apparent in the left panel of Figure 1 where the odd-in-momentum Rashba helical winding induced by the (heavily screened) external field is very small compared to the visibly prominent even-in-momentum spin texture stemming from the CrTe_2_ centrosymmetric crystal field. Achieving a Rashba term in CrTe_2_ similar to that of the Janus CrXTe would require an immense applied field  1–2 V nm^–1^ ≈ 50–100 V nm^–1^. This illustrates the remarkable SOT potential of Janus CrXTe already at this ground-state level.

The magnetization dynamics, governed by the Landau–Lifshitz–Gilbert equation, is driven by the spin–orbit torque density ***T***, which is determined by the nonequilibrium spin density ***S*** as
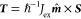
1where ***m̂*** is the magnetization direction unit vector, and *J*_ex_ is the exchange energy which couples the localized magnetic moments with those of the itinerant electrons.^1^ The exact form of the nonequilibrium response is dictated by the subjacent crystal symmetries via invariant theory.^55^ Indeed, by removing the degeneracy between both chalcogen atoms the point group symmetry is reduced from *D*_3*d*_ in CrTe_2_, where inversion symmetry forbids an SOT response, to *C*_3*v*_, which has been shown to allow for field-free PMA switching via unconventional torques.^22^ The nonequilibrium spin density, expanded up to first order, with respect to both the driving electric field () and the magnetization direction (***m̂***) reads
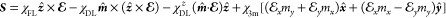
2with χ_α_ the spin linear response coefficients, and τ_α_ = ℏ^–1^*J*_ex_χ_α_ the associated spin-torque conductivity. The terms proportional to χ_FL_ and χ_DL_ represent the conventional field-like and damping-like torques respectively, while χ_DL_^*z*^ corresponds to an out-of-plane anisotropy of the damping-like torque. These torques are general to arbitrary noncentrosymmetric systems and drive the magnetization to an in-plane stationary state along the  direction. Thus, additional fields are required in order to switch a system with perpendicular magnetic anisotropy. The term proportional to χ_3m_ represents the so-called 3m torque, which is particular to systems with *C*_3*v*_ (also called 3m) symmetry. This contribution is related to a current-induced in-plane magnetic anisotropy,^56^ which modifies the stationary state of the magnetization inducing an out-of-plane instability, thus enabling field-free switching of a perpendicular magnetization.^22^ We refer the reader to section S5 in the Supporting Information for a more-complete calculation of the nonequilibrium spin density and our choices for the symmetry-based expansion.

We calculate the nonequilibrium spin density at the Fermi level (ε_F_), using the Kubo–Bastin formula,
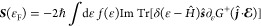
3where ***ŝ***, *Ĥ*, and ***ĵ*** are the spin, Hamiltonian, and current density operators, respectively, *f* is the Fermi–Dirac distribution, and *G*^+^ = lim_η→0_[*Ĥ* – ε + *i*η]^−1^ represents the retarded Green’s function. We numerically compute the Kubo–Bastin formula by employing a kernel polynomial method expansion, which includes the choice of a finite broadening (η = 25 meV).^57^ Our calculations are made within the broad band approximation, which suffices to compare the effect of inversion symmetry breaking mechanisms for the different systems. In order to discern the symmetry-allowed torque contributions represented in eq 2, we compute the nonequilibrium spin density in a set of 18 magnetization directions for each material and disentangle their functional form with respect to ***m̂***. Note that each of these systems requires its own ab initio and Wannier calculations as well; thus, we highlight the computational capability of the developed workflow. The exchange coupling *J*_ex_ is calculated as the average spectral difference between the spin majority and spin minority density of states.

The equilibrium electronic structure analysis of the systems foresees an enhanced SOT response in Janus systems, compared to those of the electric-field-assisted CrTe_2_. This is further confirmed and quantified by our quantum transport simulations.

An SOT response is activated in CrTe_2_ by applying a transversal electric field , as showcased for the field-like torque in Figure 2a, while all other SOT components exhibit the same tendency (see section S6 in the Supporting Information). Indeed, while the SOT response is negligible in the centrosymmetric system, it increases linearly with respect to , as seen in the inset of Figure 2a. The linear dependence persists through the entire  range explored, showing that even large symmetry-breaking applied fields up to 2 V nm^–1^ remain perturbative, with respect to the internal centrosymmetric crystal field. We observe a larger field-like torque in the hole side of the spectrum, which can be associated with larger Fermi contours closer to the Brillouin zone edges, while at the Fermi level τ_FL_ compares moderately to other two-dimensional systems, with values of the order of .^17^

**Figure 2:**
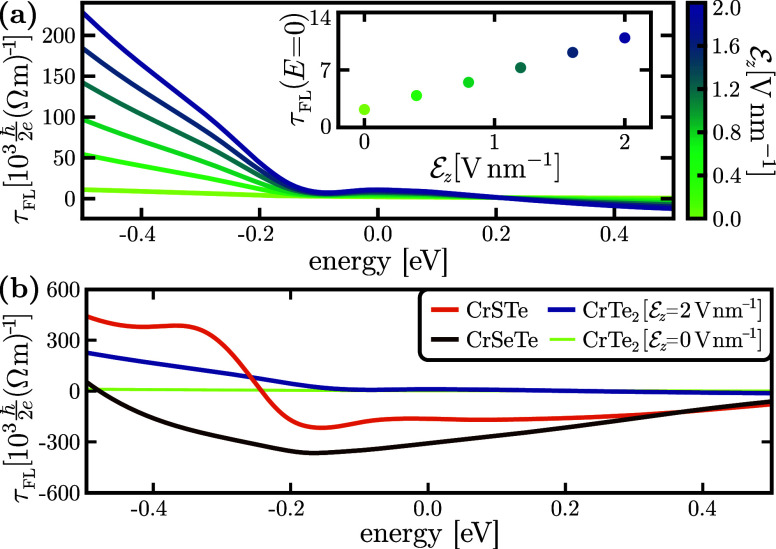
(a) Field-like spin-torque conductivity τ_FL_ in CrTe_2_, computed as a function of the transversal electric field . Inset: τ_FL_ at the Fermi level, exhibiting a linear dependence with . (b) τ_FL_ in Janus CrXTe systems, showing much larger SOT than non-Janus CrTe_2_.

Janus CrXTe systems allow us to fully achieve the potential of chromium-based TMDs for SOT applications. The strong internal electric field generated by the asymmetric crystal structure enables a field-like torque at the Fermi level of  in Janus systems, 10–100 times larger than the electric-field-assisted CrTe_2_, as shown in Figure 2b. This huge SOT enhancement is present throughout a wide energy window about the Fermi level, and holds for all torque components (see section S6 in the Supporting Information). The obtained values are comparable with the highest torques reported in two-dimensional systems, which however rely on spin transfer from a SOC material to a ferromagnet, thus being highly susceptible to the interface quality.^15,58−60^

The SOT enhancement due to structural inversion symmetry breaking comes at the expense of a modification of the ferromagnetic properties. Our ab initio calculations show that the ferromagnetic phase shifts to an in-plane magnetic anisotropy for the Janus CrXTe systems. A PMA is recovered by applying tensile strain, as shown in Figure 3a, where a prominent enhancement of the spin-torque conductivity is additionally observed. Indeed, the strain-induced magnetic anisotropy shift in CrXTe is driven by the Te atom, which presents larger SOC than its S/Se counterpart,^46,47^ thus enhancing the SOT response.

**Figure 3:**
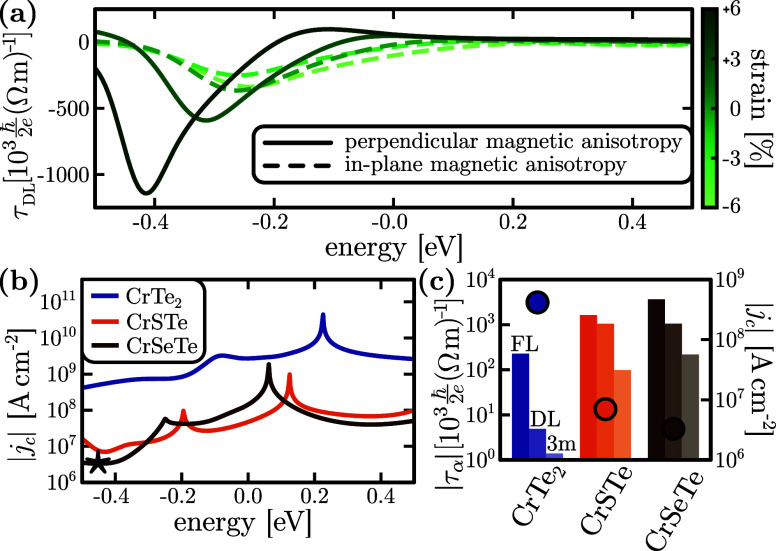
(a) τ_DL_ in CrSTe for various strain values, exhibiting PMA for strain larger than 1% (solid curves), and in-plane anisotropy otherwise (dashed curves). (b) Critical switching current *j*_c_ in Janus CrXTe (+6% strain), and in non-Janus CrTe_2_ (0% strain, and  = 2 V nm^–1^). The star marks the overall optimal switching current (*j*_c_^★^ = 3 × 10^6^ A cm^–2^). (c) Optimal switching current (right *y*-axis; filled circles) and the corresponding spin-torque conductivities (left *y*-axis; bars) for each system.

We finally showcase the enormous potential of Cr-based Janus TMDs for SOT applications by computing the critical PMA switching current. The critical switching current is estimated as^61,62^
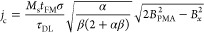
4Here, *M*_s_ is the saturation magnetization, *t*_FM_ the system’s thickness, σ the longitudinal conductivity, and β = τ_FL_/τ_DL_; with all of these quantities obtained from our ab initio and quantum transport results. For the remaining parameters—namely, the Gilbert damping (α = 0.01) and the perpendicular anisotropy field (*B*_PMA_ = 0.1 T)—we choose reasonable constant values, noting that they are fairly tunable using experimental conditions.^63,64^ Finally, *B*_*x*_ represents an in-plane effective magnetic field that drives the system out of the in-plane stationary state along  (promoted by the field-like and damping-like torques), allowing PMA switching. In conventional systems, an applied external magnetic field *B*_*x*_ is required;^65^ however, the 3m torque serves this purpose in CrXTe, allowing for field-free switching of a perpendicular magnetic state.^22^ The existence of a nonzero 3m torque, which we find roughly of the order of ∼10% of the damping-like torque throughout most of the explored energy range (see section S6 in the Supporting Information), thus stands out as one of the most remarkable features of the studied Cr-based Janus materials, allowing for a truly all-in-one SOT platform without the need for external magnetic nor electric fields, surpassing previous proposals.^43,66,67^

Because *j*_c_ gathers multiple magnetic and transport properties, it serves as an ultimate SOT figure of merit, providing a direct grasp of the power efficiency gain. The potential of Janus CrXTe systems is once again manifested, achieving critical switching currents 10–100 times smaller than those of non-Janus CrTe_2_, as shown in Figure 3b. The reduction of the switching current is indeed the result of enhanced SOTs in the Janus systems, evidenced in Figure 3c, which shows τ_FL_, τ_DL_ and τ_3m_ at the optimal *j*_c_ energy value for each system. Moreover, we note that the critical switching currents calculated by eq 4 correspond to an upper bound for the real values, as it is derived within a macrospin approximation, whereas experimental evidence indicates that the switching occurs via domain wall nucleation and propagation.^22,68^ We find an overall optimal switching current of *j*_c_^★^ = 3 × 10^6^ A cm^–2^, occurring for CrSeTe with +6% strain at 0.4 eV below the Fermi level. Experimentally, such strain can appear naturally by the substrate or the growth conditions (see section S3 in the Supporting Information). This value is already highly competitive among van der Waals magnetic materials,^69−76^ where the reported critical switching currents range from 5 × 10^5^ A/cm^2^ to 2.5 × 10^7^ A/cm^2^, respectively, as reported in Cr_2_Ge_2_Te_6_/Ta^69^ and Fe_3_GeTe_2_/Pt^70^ heterostructures, while experimental conditions may result in further reduction of the critical current.

Altogether, we have found that magnetic chromium-based Janus TMDs offer a remarkable SOT performance. Concatenating ab initio and quantum transport methodologies, we have shown that the large SOT response stems from huge internal electric fields due to their asymmetric crystal structure, yielding a competitive switching current with the additional advantage of neither requiring assistance of external fields nor the transmission of spin current through an imperfect interface. Such results present magnetic Janus TMDs as efficient materials for designing ultimate SOT-MRAM technologies.

## Supporting Information Available

Details of the ab initio methodology, the calculated internal electric fields, motivation for strain-dependent calculations, Wannier models accuracy, and complete results of the Kubo–Bastin spin–orbit torque calculations (PDF)
