# Supplementary material for: Field-Free Spin–Orbit Torque Switching in Janus Chromium Dichalcogenides
Source: Nano Lett. 2024 Sep 13;24(38):11889–94. doi: 10.1021/acs.nanolett.4c03029 (PMC11440640; doi:10.1021/acs.nanolett.4c03029)
Supplement: Supplementary file 1 — nl4c03029_si_001.pdf [file nl4c03029_si_001.pdf]

Supporting information

Field-free spin-orbit torque switching in Janus chromium dichalcogenides

Libor Vojáček,[1,*] Joaquín Medina Dueñas,[2,3,†] Jing Li,[4] Fatima Ibrahim,[1] Aurélien Manchon,[5] Stephan Roche,[2,6] Mairbek Chshiev,[1,7] and José H. García[2]

[1] *Université Grenoble Alpes, CEA, CNRS, IRIG-Spintec, 38000 Grenoble, France*
[2] *ICN2 — Institut Català de Nanociència i Nanotecnologia, CSIC and BIST, Bellaterra, 08193 Barcelona, Spain*
[3] *Universitat Autònoma de Barcelona (UAB), Bellaterra, 08193 Barcelona, Spain*
[4] *Université Grenoble Alpes, CEA, Leti, F-38054, Grenoble, France*
[5] *Aix-Marseille Université, CNRS, CINAM, Marseille 13288, France*
[6] *ICREA — Institució Catalana de Recerca i Estudis Avançats, 08010 Barcelona, Spain*
[7] *Institut Universitaire de France, 75231 Paris, France*

CONTENTS

S1. Ab initio calculation details 2
S1.1. The spin operator in real space 2

S2. Electrostatics of CrXTe 2
S2.1. Internal E-fields in CrXTe monolayers 3
S2.2. Relative permittivity of $CrTe_2$ monolayer 3
S2.3. The equivalent external E-field to the Janus internal E-field 3

S3. Strain (lattice parameter variation) 3

S4. Quality of the Wannier models 5
S4.1. Quality convergence with the Wannier supercell size 5
S4.2. Quality of the final models 6

S5. Non-Equilibrium Spin Density and Symmetry-Allowed Contributions 6

S6. Transport simulations: complete results 7
S6.1. Strainless 7
S6.2. Strain-dependent 8

References 9

S1. AB INITIO CALCULATION DETAILS

The *ab initio* ground state calculations were performed using density functional theory (DFT) as implemented in the Vienna *ab initio* simulation package (`VASP`) [S1, S2] with the GGA-PBE exchange-correlation functional [S3]. The Hubbard U correction in Dudarev's formulation [S4] was applied to the Cr d orbitals with an effective Hubbard correction $U_{\text{eff}} = 3.0$ eV, a value that makes the band structure closely match the experimentally-measured one [S5]. A Cr pseudopotential with semicore p electrons was chosen and an energy cutoff of 330 eV was used for the plane-wave basis. Brillouin zone was sampled with a 19x19x1 Γ-centered mesh. The van der Waals interaction was approximated by the DFT-D3 method [S6] with the Becke-Johnson damping [S7]. Forces were minimized below 10^{-3} eV/Å during the full structural relaxation and total energy below 10^{-7} eV to achieve the electronic self-consistency. Spin-orbit coupling was included except in the relaxation step.

The Wannier tight-binding models were constructed using the `Wannier90` package, with a 22-orbital basis (10 Cr-d and 2×6 chalcogen-p orbitals) and a large 25×25 real-space supercell that limits the real-space interactions. A smaller supercell might very well be sufficient, see Sec. S4 S4.2. The *Wannier* energy window was chosen to tightly encompass the 22 bands with the Cr-d and chalcogen-p character. The *frozen* energy window has an upper limit to avoid including an intersecting parasitic s/dz^2 band and keeps an additional margin of 0.2 eV from this band, a value chosen to optimize the RMSE quality of the $CrTe_2$ Wannier model (see Sec. S4). Disentanglement and maximal localization were performed with 200 and 100 maximum steps, respectively. The spin operator in the maximally-localized Wannier function (MLWF) basis was derived, as described below.

S1.1. The spin operator in real space

Accessing the spin expectation values of the interpolated band structure with Wannier tight-binding models requires deriving the Wannier real-space spin operator.

Just like any other operator that can be obtained from the *ab initio* calculation (for all the *ab initio* k-points $\boldsymbol{q}$), the *spin operator* in the basis of *ab initio* eigenstates $\mathcal{S}^{\text{H}}_{mn}(\mathbf{q})$ can be converted into its real-space representation by first applying the semi-unitary matrix $V_{m'm}(\mathbf{q})$ to convert $\mathcal{S}$ from the *Hamiltonian* gauge to the *Wannier* gauge

$$\mathcal{S}^{\text{W}}_{mn}(\boldsymbol{q}) = \sum_{m'n'} V^{\dagger}_{m'm}(\boldsymbol{q}) \cdot \mathcal{S}^{\text{H}}_{m'n'}(\mathbf{q}) \cdot V_{n'n}(\boldsymbol{q}), \tag{S1}$$

where the spin operator $\hat{\mathcal{S}}$ projected onto the basis of the *ab initio* eigenstates ψ in its matrix form $\mathcal{S}^{\text{H}}_{m'n'}(\mathbf{q}) = \left\langle \psi_{m'\boldsymbol{q}} | \hat{\mathcal{S}} | \psi_{n'\boldsymbol{q}} \right\rangle$ is obtained with the help of the `vaspspn` module of `WannierBerri` [S8]. Primed indices run over the (larger) space of disentanglement bands.

Follows the direct Fourier sum over the *ab initio* grid

$$\mathcal{S}_{mn}(\boldsymbol{R}) \equiv \frac{1}{N_{\boldsymbol{q}}} \sum_{\boldsymbol{q}} \mathcal{S}^{\text{W}}_{mn}(\boldsymbol{q}) \cdot e^{-i\boldsymbol{q}\cdot\boldsymbol{R}}, \tag{S2}$$

with $N_{\boldsymbol{q}}$ the number of *ab initio* grid points $\boldsymbol{q}$.

Having $\mathcal{S}_{mn}(\boldsymbol{R})$ at hand, its interpolation $\overline{\mathcal{S}}^{\text{H}}_{mn}(\boldsymbol{k})$ to an *arbitrary* k-vector $\boldsymbol{k}$ involves an inverse Fourier sum $\overline{\mathcal{S}}^{\text{W}}_{mn}(\boldsymbol{k}) = \sum_{\mathbf{R}} \mathcal{S}_{mn}(\boldsymbol{R}) \cdot e^{i\boldsymbol{k}\cdot\boldsymbol{R}}$ over the real-space lattice vectors $\boldsymbol{R}$ followed by a rotation back to the Hamiltonian gauge of the original eigenstates $\overline{\mathcal{S}}^{\text{H}}_{mn}(\boldsymbol{k}) = (U^{\dagger} \cdot \mathcal{S}^{\text{W}} \cdot U)_{mn}$, where U_{mn} is a unitary matrix which diagonalizes the interpolated Hamiltonian $\overline{\mathcal{H}}^{\text{W}}_{mn}(\boldsymbol{k}) = \sum_{\mathbf{R}} \mathcal{H}_{mn}(\boldsymbol{R}) \cdot e^{i\boldsymbol{k}\cdot\boldsymbol{R}}$.

The described procedure is implemented in the python package `spinWannier` [S9].

S2. ELECTROSTATICS OF CrXTe

The field-like torque is directly linked to the Rashba effect attributed to an inversion symmetry breaking, which comes from an electrical dipolar field across the monolayer plane.

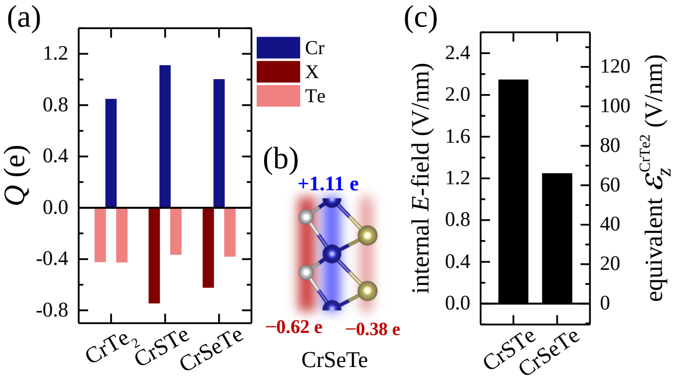

FIG. S1. Electrostatics of CrXTe monolayers. (a,b) The (Bader) charge transfer Q is different at the X and Te sides, giving rise to (c) a large internal electric field ~ 2 V/nm. The equivalent out-of-plane E-field over $CrTe_2$ would have to be an enormous $\epsilon_{\mathrm{CrTe2}}\, 2$ V/nm ~ 100 V/nm, demonstrating the potency of CrXTe.

S2.1. Internal E-fields in CrXTe monolayers

The spontaneous internal E-fields in the Janus CrXTe come from the charge imbalance, as shown in Fig. S1. This creates a work function difference and an internal electric field $\sim$ 1–2 V/nm in CrXTe. We calculate these values from the work function difference $\Delta\phi$ [Fig. S2(a)] divided by the thickness of the layer, which is derived from the positions of the chalcogen atom centers extended by their Wigner-Seitz radii.

S2.2. Relative permittivity of $CrTe_2$ monolayer

Subtracting the xy-averaged potential of the $CrTe_2$ monolayer under an out-of-plane E-field of 2 V/nm and under no E-field, we can estimate its relative permittivity from the gradient of the potential inside the layer and in vacuum, see Fig. S2. In vacuum, the potential gradient corresponds to the imposed $E_{\mathrm{ext}} = 2$ V/nm, which is largely screened inside $CrTe_2$ to $E_{\mathrm{ind}} = 0.038$ V/nm. The $E_{\mathrm{ind}}/E_{\mathrm{ext}}$ ratio gives the relative permittivity of $CrTe_2$, $\epsilon_{\mathrm{CrTe2}} = 52.9$. This is a prefactor that should be applied to the *internal* electric fields of CrXTe

$$\mathcal{E}_{\mathrm{ext}}^{\mathrm{equivalent\ for\ CrTe2}} = \epsilon_{\mathrm{CrTe2}}\, \mathcal{E}_{\mathrm{int}}^{\mathrm{CrXTe}} \tag{S3}$$

to get an equivalent *external* E-field over $CrTe_2$ to achieve a comparable Rashba splitting.

S2.3. The equivalent external E-field to the Janus internal E-field

In Tab. I, the ratio between χ_{FL} of CrXY and gated $CrTe_2$ gives an estimation of the required E_{ext} which should be applied to $CrTe_2$ to obtain a similar χ_{FL} in $CrTe_2$ as are spontaneously present in CrXY. The values are colossal 30 to 60 V/nm. They correspond well to a simple multiplication of the CrXY internal E-fields E_{int} with the relative permittivity of $CrTe_2$ $\epsilon_{\mathrm{CrTe2}} = 52.9$, giving a 113 and 66 V/nm.

S3. STRAIN (LATTICE PARAMETER VARIATION)

Although theoretical studies suggest that the in-plane lattice parameter of $CrTe_2$ is reduced in its monolayer form compared to bulk [S10], experimental measurements [S5, S11] suggest that ultrathin films of $CrTe_2$ retain their lattice constant reasonably close to bulk. The experimentally measured lattice parameter of ultrathin $CrTe_2$ films $a = 3.77$ Å [S11] is $\approx 4\%$ larger than our calculated monolayer value, as shown in Fig. S3(a). Since Janus CrXTe monolayers have not yet been fabricated and their experimental structure may also slightly differ from our *ab initio* predictions, we deem it necessary to provide our results as a function of *strain*, which is not necessarily a physical strain, but rather a variation of the lattice parameter with respect to the predicted *ab initio* value.

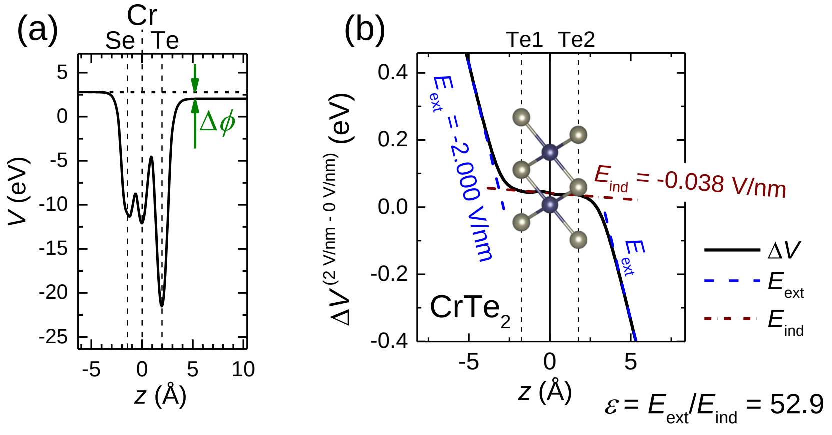

FIG. S2. Electrostatics of CrXTe. (a) The (xy-averaged) potential in CrSeTe is different at the Se and Te sides. This work function difference translates into an internal electric dipole. (b) The relative permittivity of $CrTe_2$ monolayer can be calculated from the difference between the xy-averaged potential of $CrTe_2$ monolayer under an external $E_{\text{ext}} = 2$ V/nm and without an external E-field. The potential gradient corresponds to the E-field magnitude. The ratio of the E-field outside (in vacuum) and inside the layer corresponds to ϵ_{CrTe2}.

TABLE I. The calculated internal E-fields E_{int} in CrXY monolayers and an estimated equivalent external field $E_{\text{ext}}^{\text{CrTe2}} \simeq \epsilon_{\text{CrTe2}} \cdot E_{\text{int}}$ that should act on $CrTe_2$ to reach a similar effect. Further, the calculated field-like torques χ_{FL} in Janus CrXY monolayers and an estimated external E-field that should act on $CrTe_2$ to reach similar torque magnitudes. Both these estimations result in huge $E_{\text{ext}}^{\text{CrTe2}} \approx 30$ to 50 V/nm, illustrating the superiority of Janus CrXY for producing the Rashba effect and field-like torques.

	E_{int}	$E_{\text{ext}}^{\text{CrTe2}} \simeq \epsilon_{\text{CrTe2}} \cdot E_{\text{int}}$	χ_{FL}	$E_{\text{ext}}^{\text{CrTe2}} \simeq 2.0 \cdot \chi_{\text{FL}}/\chi_{\text{FL}}^{\text{CrTe2 @ 2.0 V/nm}}$
	V/nm	V/nm	$10^3\hbar/2\text{e (Ohm m)}^{-1}$	V/nm
CrSTe	2.14	**113**	163	**30**
CrSeTe	1.25	**66**	308	**57**

$\epsilon_{\text{CrTe2}} = 52.9$; $\chi_{\text{FL}}^{\text{CrTe2 @ 2.0 V/nm}} = 10.9\ 10^3\hbar/2\text{e (Ohm m)}^{-1}$

As shown in Fig. S3(b-c), the consequence of such *positive* strain is an increase in both the effective magnetic anisotropy and the Heisenberg exchange coupling, linked to the Curie temperature.

The appropriate effective magnetic anisotropy in case of 2D magnets [S12]

$$K_{\text{eff}} = K_{\text{u}} + Z(J_z - J_x) + E_{\text{demag}} \tag{S4}$$

is a sum of the calculated single-ion anisotropy K_{u}, the (super)exchange interaction anisotropy $Z(J_z - J_x)$ with the number of nearest neighbors $Z = 6$ and the demagnetizing energy $E_{\text{demag}} = -\mu_0 M_{\text{s}}^2/2$ with its small value of ≈ -0.07 and -0.06 mJ/m^2 for CrSTe and CrSeTe respectively.

Note that the dominant contribution is expected to be the anisotropic superexchange $Z(J_z - J_x)$, while the single-ion contribution K_{u} should be negligible [S12].

All these quantities can be calculated from the *ab initio* total energies, considering the XXZ Hamiltonian [S12]

$$H = -K_u \sum_i (S_i^z)^2 - \frac{J_x}{2} \sum_{i \neq j}\sum \left(S_i^x S_j^x + S_i^y S_j^y\right) - \frac{J_z}{2} \sum_{i \neq j}\sum \left(S_i^z S_j^z\right) \tag{S5}$$

where i runs over all Cr atoms and j runs over all of their 6 nearest neighbors (thereby double counting is present and redeemed by the factor $\frac{1}{2}$ coming with J). Constructing a 2x1x1 supercell [see the inset of Fig. S3(b)] of the CrXTe unit cells with both ferromagnetic (FM) and antiferromagnetic (AF) configurations along both the x and z directions, it follows from Eq. S5 that the *ab initio* ground state energies are $E_x^{\text{FM}} = E_0 - 6S^2 J_x$, $E_x^{\text{AF}} = E_0 + 2S^2 J_x$, $E_z^{\text{FM}} = E_0 - 2S^2 K_{\text{u}} - 6S^2 J_z$ and $E_z^{\text{AF}} = E_0 - 2S^2 K_{\text{u}} + 2S^2 J_z$, from which

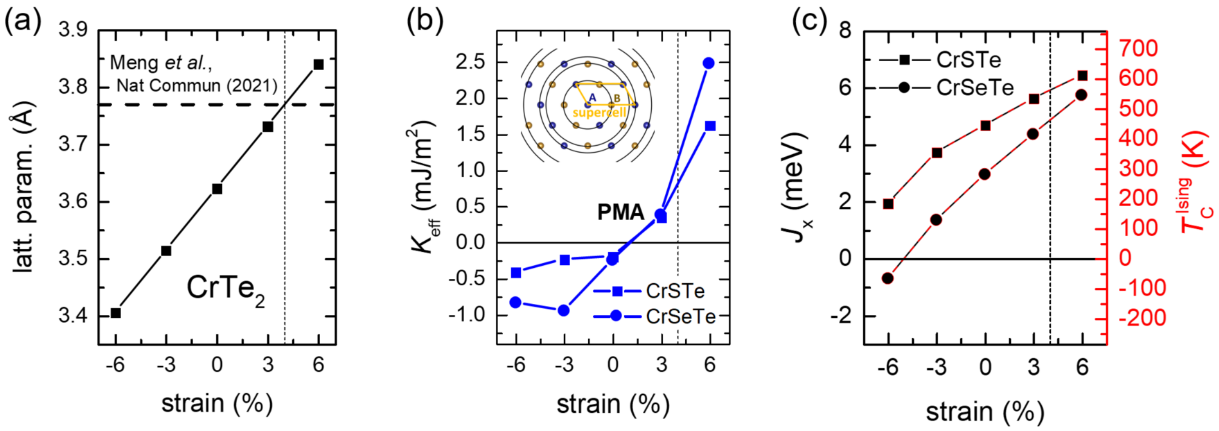

FIG. S3. The magnetic anisotropy and exchange coupling with lattice parameter variation ("strain"). 0% corresponds to the *ab initio*-predicted lattice constant. (a) The experimental lattice parameter of $CrTe_2$ [S11] is $\approx 4\%$ larger than the *ab initio* prediction. Both (b) the effective uniaxial anisotropy and (c) the nearest-neighbor exchange interaction (and the consequent Ising Curie temperature) largely increase for higher lattice parameters in CrXTe. The vertical dashed line corresponds to the $\approx 4\%$ latt. param. difference that might be expected from the comparison of DFT and experiment of $CrTe_2$ from (a). Inset of (b) shows the 2x1x1 supercell used for the Heisenberg exchange parameter calculation.

$$
\begin{aligned}
J_{x(z)} &= \left(-E^{\mathrm{FM}}_{x(z)} + E^{\mathrm{AF}}_{x(z)}\right)/8S^2\,, \\
K_{\mathrm{u}} &= \left(E^{\mathrm{FM}}_{x} + 3E^{\mathrm{AF}}_{x} - E^{\mathrm{FM}}_{z} - 3E^{\mathrm{AF}}_{z}\right)/8S^2\,,
\end{aligned}
\tag{S6}
$$

where $S = 3/2$ is the magnitude of the Cr spin [S12].

In Fig. S3(c) we also plot the strain-enhanced Curie temperature in the Ising limit [S13]

$$
T_{\mathrm{C}}^{\mathrm{Ising}} = \frac{S^2 \tilde{T}_{\mathrm{c}}}{k_{\mathrm{B}}} J_x\,,
\tag{S7}
$$

where the constant dimensionless $\tilde{T}_{\mathrm{c}} = 3.64$ for a hexagonal lattice [S13], k_{B} is the Boltzmann constant.

S4. QUALITY OF THE WANNIER MODELS

We provide a quantitative estimate of the quality of the derived Wannier tight-binding models, which aim to accurately describe the *ab initio* band energies and spin expectation values. For this, the band structure along the K−Γ−M − K with 101 points along each segment is calculated both from *ab initio* and the derived Wannier TB model and compared to obtain the RMSE errors as

$$
\mathrm{RMSE}(E) = \sqrt{\frac{1}{N} \sum_{\langle \mathrm{kpath} \rangle} \left(E^{\mathrm{DFT}} - E^{\mathrm{WannTB}}\right)^2}
\tag{S8}
$$

and analogically for the spin expectation values. N is the number of summed data points. The averaging is performed over an energy window encompassing the whole Fermi sea $\approx E_{\mathrm{F}} - 5$ eV and up to $\approx E_{\mathrm{F}} + 4$ eV.

S4.1. Quality convergence with the Wannier supercell size

The real-space interactions need to be limited by a large enough supercell to converge the RMSE below a chosen threshold, as plotted in Fig. S4. A 25×25 supercell provides sufficient accuracy. The saturation of the *RMSE of energy* for CrXTe indicates a (small) constant offset of the whole energy spectrum due to slightly (≈ 1 meV) misaligned Fermi energy values.

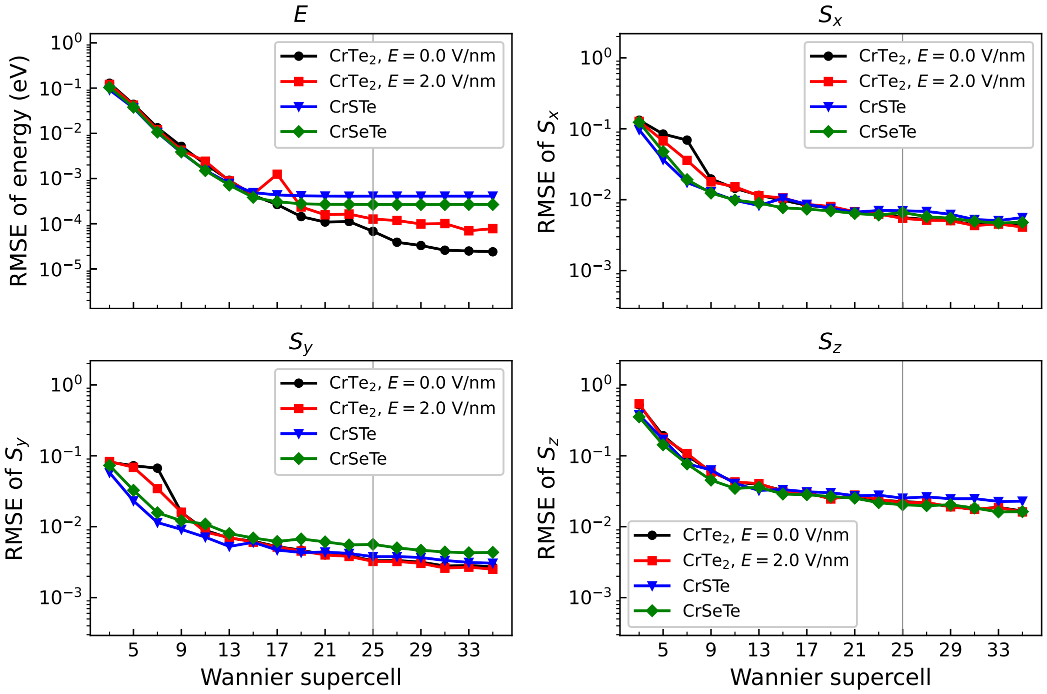

FIG. S4. The root mean square error of energy and spin expectation values of the Wannier TB model vs. the original DFT calculation, averaged along the standard $\mathrm{K}-\Gamma-\mathrm{M}-\mathrm{K}$ k-path and within an energy window from $\approx E_\mathrm{F}-5$ eV up to $\approx E_\mathrm{F}+4$ eV. The RMSE of energy exponentially decreases with increasing Wannier supercell (interaction cutoff). The vertical line denotes the 25×25 supercell, used for further transport calculations.

S4.2. Quality of the final models

Figure S5 shows the RMSE of energy and spin for $CrTe_2$ (with and without external E-field), CrSTe, and CrSeTe calculated with different *ab initio* magnetization directions $\vec{m} = (\sin\theta\cos\phi, \sin\theta\sin\phi, \sin\theta)$. The errors are fairly small: ≈ 1 meV for energy and ≈ 0.03 for spin expectation values.

S5. NON-EQUILIBRIUM SPIN DENSITY AND SYMMETRY-ALLOWED CONTRIBUTIONS

Consistent with the linear transport regime, we expand the non-equilibrium spin density $\boldsymbol{S}$ to linear order in terms of its $\mathcal{E}_j$ $(j=x,y)$ dependence. On the other hand, since the magnetization direction does not constitute a perturbative parameter, we expand $\boldsymbol{S}$ up to second order with respect to $\hat{\boldsymbol{m}}$. We express the non-equilibrium spin density as $\boldsymbol{S}=\boldsymbol{S}_0+\boldsymbol{S}_{3\mathrm{m}}$, where the first term groups the torque contributions which are allowed in arbitrary non-centrosymmetric systems, while the second represents torque contributions enabled by the reduced 3m symmetry. The first contribution reads

$$\boldsymbol{S}_0 = \chi_\mathrm{FL}[1-\xi_\mathrm{FL}(m_x^2+m_y^2)]\hat{\boldsymbol{z}}\times\boldsymbol{\mathcal{E}} - \chi_\mathrm{DL}\hat{\boldsymbol{m}}\times(\hat{\boldsymbol{z}}\times\boldsymbol{\mathcal{E}}) - \chi_\mathrm{DL}^z(\hat{\boldsymbol{m}}\cdot\boldsymbol{\mathcal{E}})\hat{\boldsymbol{z}} - \chi_\parallel[\hat{\boldsymbol{m}}\cdot(\hat{\boldsymbol{z}}\times\boldsymbol{\mathcal{E}})]\hat{\boldsymbol{m}} - \chi_\parallel^z[\hat{\boldsymbol{m}}\cdot(\hat{\boldsymbol{z}}\times\boldsymbol{\mathcal{E}})]m_z\hat{\boldsymbol{z}}\ , \tag{S9}$$

showing that all systems allow for many contributions beyond the conventional field-like and damping-like torques [?], respectively represented in the terms proportional to χ_FL and χ_DL, where the field-like torque is modulated by the total in-plane magnetization via ξ_FL. χ_DL^z accounts for an anisotropic damping of the in-plane and out-of-plane magnetization components towards the steady state along $\hat{\boldsymbol{z}}\times\boldsymbol{\mathcal{E}}$. $\chi_\parallel$ represents a non-equilibrium spin density component parallel to the magnetization which cannot exert torque, while $\chi_\parallel^z$ corresponds to an out-of-plane anisotropy of the aforementioned component which, along with the field-like torque, induces an elliptic precession of the magnetization about $\hat{\boldsymbol{z}}\times\boldsymbol{\mathcal{E}}$. A set of unconventional torques are additionally enabled by the system's 3m

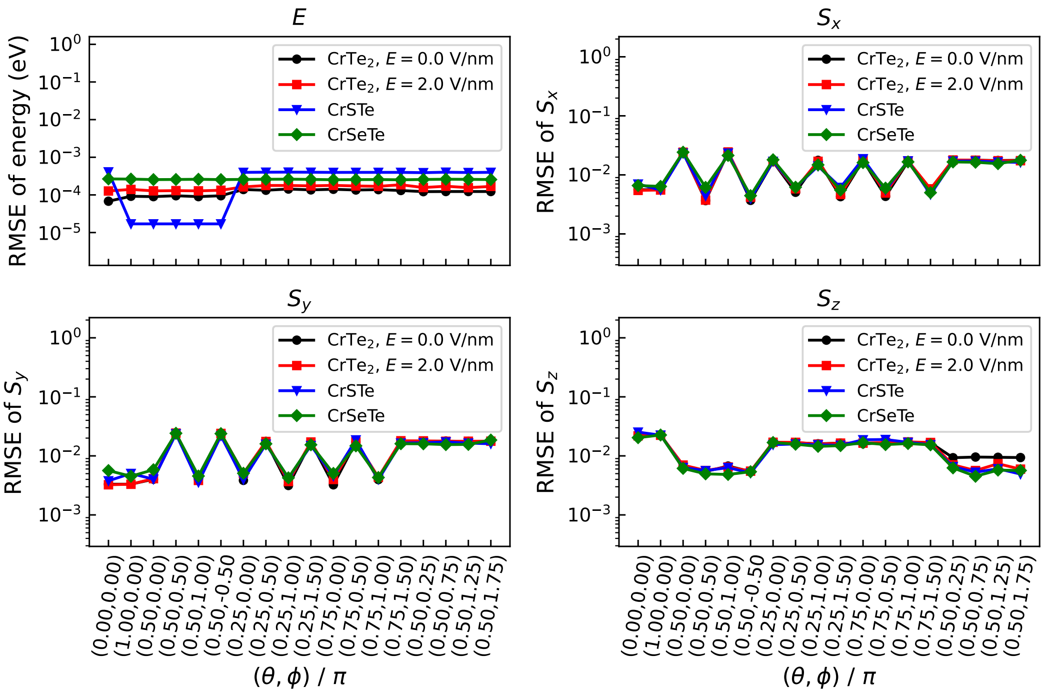

FIG. S5. The root mean square error of energy and spin expectation values of the Wannier TB model vs. the original DFT calculation, averaged along the standard K$-\Gamma-$M $-$ K k-path and within an energy window from $\approx E_{\mathrm{F}} - 5$ eV up to $\approx E_{\mathrm{F}} + 4$ eV. The azimuthal and polar angles θ and ϕ denote calculations with different magnetization directions. The errors are small: around 1 meV in energy and 0.01 in spin expectation values.

symmetry, which are represented in

$$\begin{aligned}\boldsymbol{S}_{3\mathrm{m}} =& \chi_{3\mathrm{m}}[(\mathcal{E}_x m_y + \mathcal{E}_y m_x)\hat{\boldsymbol{x}} + (\mathcal{E}_x m_x - \mathcal{E}_y m_y)\hat{\boldsymbol{y}}] - \chi^{(2)}_{3\mathrm{m}} m_z[(-\mathcal{E}_x m_x + \mathcal{E}_y m_y)\hat{\boldsymbol{x}} + (\mathcal{E}_x m_y + \mathcal{E}_y m_x)\hat{\boldsymbol{y}}] \\ &+ \chi^{z}_{3\mathrm{m}}(\mathcal{E}_x m_x^2 - \mathcal{E}_x m_y^2 - 2\mathcal{E}_y m_x m_y)\hat{\boldsymbol{z}} \ .\end{aligned} \tag{S10}$$

The term proportional to $\chi_{3\mathrm{m}}$ represents the so-called 3m torque, while the other second-order terms stem from a contribution of the form $\hat{\boldsymbol{m}} \times \boldsymbol{s}_{3\mathrm{m}}$, with $\boldsymbol{s}_{3\mathrm{m}}$ the aforementioned first order 3m spin density. The 3m torque derives from a current-induced in-plane magnetic anisotropy which, when applying a driving electric field $\boldsymbol{\mathcal{E}} = \mathcal{E}_x \hat{\boldsymbol{x}}$ perpendicular to the mirror plane, lies along the $m_x = -m_y$ axis [S14]. Thus, the first order 3m torque $\chi_{3\mathrm{m}}$ accounts for precession about the anisotropy axis, while the second order torques $\chi^{(2)}_{3\mathrm{m}}$ and $\chi^{z}_{3\mathrm{m}}$ account for a damping parallel to it.

We note that in the chosen basis for the expansion of the non-equilibrium spin density, the first order contributions already capture the SOT response in both the equilibrium configuration $\hat{\boldsymbol{m}} = \pm\hat{\boldsymbol{z}}$ and the current-induced stationary state $\hat{\boldsymbol{m}} \parallel \hat{\boldsymbol{z}} \times \boldsymbol{\mathcal{E}}$, while the second and higher order contributions only represent corrections at other intermediate magnetization directions.

S6. TRANSPORT SIMULATIONS: COMPLETE RESULTS

S6.1. Strainless

We present the complete spin-orbit torque results for all the studied systems, at 0% strain. In order to disentangle the symmetry-allowed torque contributions we compute the non-equilibrium spin density for a set of 18 magnetization directions for each system, consisting of 8 evenly spaced φ points for $\theta = \pi/2$, 4 evenly spaced φ points for $\theta = \pi/4, 3\pi/4$, and the two poles $\theta = 0, \pi$, with (θ, φ) the magnetization polar and azimuthal angles. The spin-torque conductivities are shown in Fig. S6. We observe that all torque contributions are much larger in the Janus structures than in the electric field assisted $CrTe_2$, while they are negligible in centrosymmetric $CrTe_2$. We note that the SOT response in centrosymmetric $CrTe_2$ with $\mathcal{E}_z = 0$ is non-zero, which derives from the numerical approximations

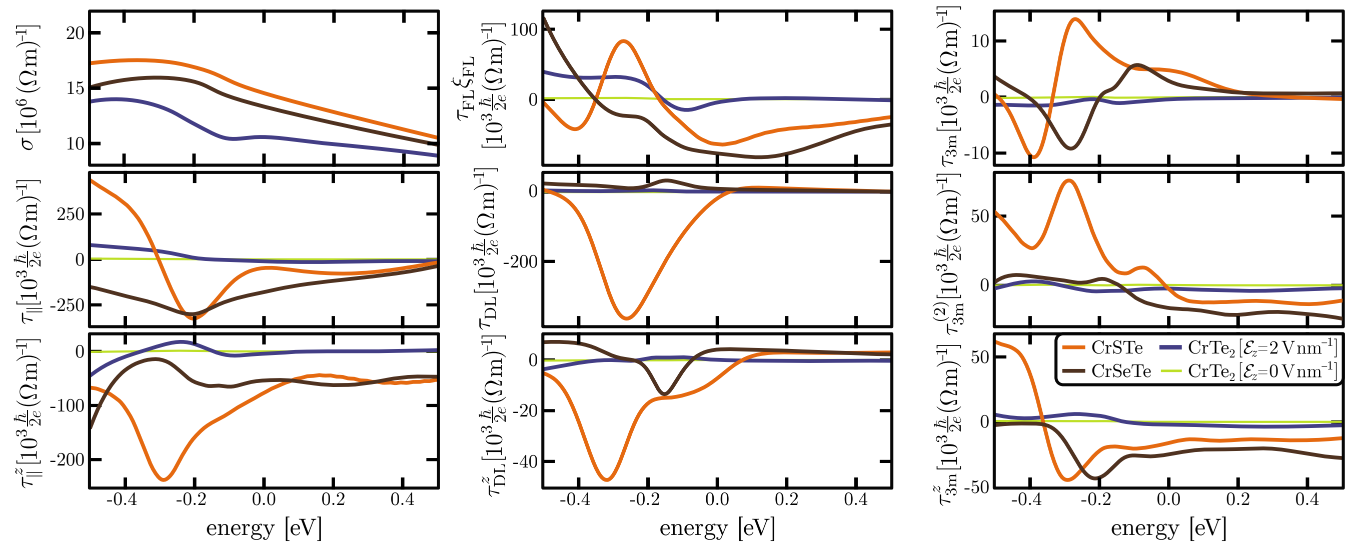

FIG. S6. Longitudinal conductivity σ, in units of $10^6(\Omega\,\mathrm{m})^{-1}$, and all spin-torque conductivities τ_α, in units of $10^3\,\frac{\hbar}{2e}(\Omega\,\mathrm{m})^{-1}$, for all systems. The longitudinal conductivity of $CrTe_2$ is unaffected by $\mathcal{E}_z$

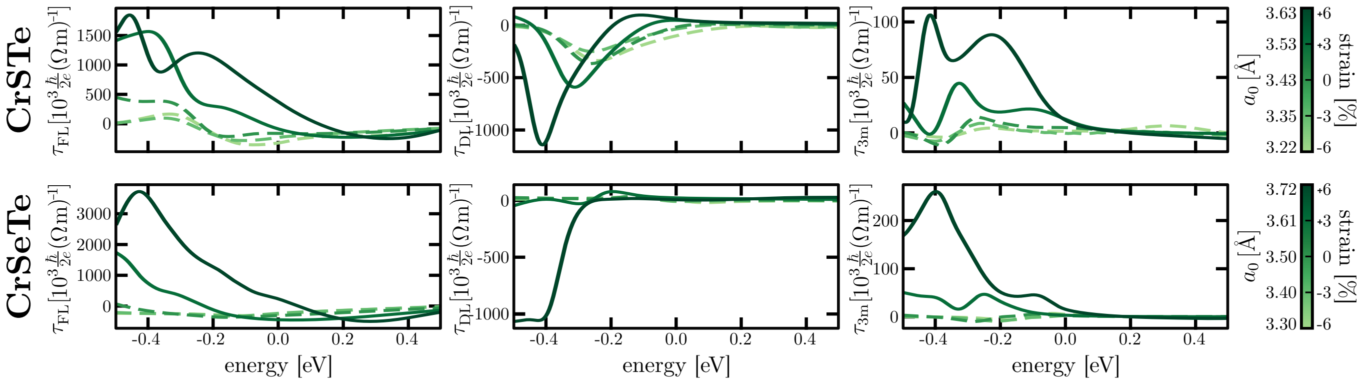

FIG. S7. Field-like, damping-like and 3m spin-torque conductivities, in units of $10^3\,\frac{\hbar}{2e}(\Omega\,\mathrm{m})^{-1}$ as a function of strain. Solid and dashed curves correspond to perpendicular and in-plane magnetic anisotropy respectively. The corresponding lattice parameter a_0 is indicated in the colorbar.

performed during the Wannierization procedure and lie within its numerical precision; yet it is negligible compared to the $\mathcal{E}_z \neq 0$ systems. Additionally, the results for the longitudinal conductivity show that it remains of the same order in Janus vs non-Janus systems.

S6.2. Strain-dependent

Fig. S7 shows the field-like, damping-like and 3m torques as a function of strain. All torques are enhanced with strain, retrieving a perpendicular magnetic anisotropy for strain $\geq 1\%$.

* libor.vojacek@cea.fr
† joaquin.medina@icn2.cat

[S1] G. Kresse and J. Hafner, "*Ab initio* molecular dynamics for liquid metals," Physical Review B **47**, 558–561 (1993).
[S2] G. Kresse and J. Furthmüller, "Efficiency of ab-initio total energy calculations for metals and semiconductors using a plane-wave basis set," Computational Materials Science **6**, 15–50 (1996).
[S3] John P. Perdew, Kieron Burke, and Matthias Ernzerhof, en"Generalized Gradient Approximation Made Simple," Physical Review Letters **77**, 3865–3868 (1996).
[S4] S. L. Dudarev, G. A. Botton, S. Y. Savrasov, C. J. Humphreys, and A. P. Sutton, en"Electron-energy-loss spectra and the structural stability of nickel oxide: An LSDA+U study," Physical Review B **57**, 1505–1509 (1998).
[S5] Xiaoqian Zhang, Qiangsheng Lu, Wenqing Liu, Wei Niu, Jiabao Sun, Jacob Cook, Mitchel Vaninger, Paul F. Miceli, David J. Singh, Shang-Wei Lian, Tay-Rong Chang, Xiaoqing He, Jun Du, Liang He, Rong Zhang, Guang Bian, and Yongbing Xu, "Room-temperature intrinsic ferromagnetism in epitaxial CrTe2 ultrathin films," Nature Communications **12**, 2492 (2021).
[S6] Stefan Grimme, Jens Antony, Stephan Ehrlich, and Helge Krieg, en"A consistent and accurate *ab initio* parametrization of density functional dispersion correction (DFT-D) for the 94 elements H-Pu," The Journal of Chemical Physics **132**, 154104 (2010).
[S7] Stefan Grimme, Stephan Ehrlich, and Lars Goerigk, en"Effect of the damping function in dispersion corrected density functional theory," Journal of Computational Chemistry **32**, 1456–1465 (2011).
[S8] Stepan S. Tsirkin, en"High performance Wannier interpolation of Berry curvature and related quantities with Wannier-Berri code," npj Computational Materials **7**, 33 (2021).
[S9] Libor Vojáček, "spinWannier: python package," (2024).
[S10] Yuhang Liu, Sohee Kwon, George J. de Coster, Roger K. Lake, and Mahesh R. Neupane, "Structural, electronic, and magnetic properties of CrTe 2," Physical Review Materials **6**, 084004 (2022).
[S11] Lingjia Meng, Zhang Zhou, Mingquan Xu, Shiqi Yang, Kunpeng Si, Lixuan Liu, Xingguo Wang, Huaning Jiang, Bixuan Li, Peixin Qin, Peng Zhang, Jinliang Wang, Zhiqi Liu, Peizhe Tang, Yu Ye, Wu Zhou, Lihong Bao, Hong-Jun Gao, and Yongji Gong, en"Anomalous thickness dependence of Curie temperature in air-stable two-dimensional ferromagnetic 1T-CrTe2 grown by chemical vapor deposition," Nature Communications **12**, 809 (2021).
[S12] J L Lado and J Fernández-Rossier, "On the origin of magnetic anisotropy in two dimensional CrI $_3$," 2D Materials **4**, 035002 (2017).
[S13] Daniele Torelli and Thomas Olsen, en"Calculating critical temperatures for ferromagnetic order in two-dimensional materials," 2D Materials **6**, 015028 (2018).
[S14] Oyvind Johansen, Vetle Risinggard, Asle Sudbo, Jacob Linder, and Arne Brataas, "Current Control of Magnetism in Two-Dimensional Fe_3GeTe_2," Phys. Rev. Lett. **122**, 217203 (2019).
